# Dapagliflozin Mitigates Doxorubicin-Caused Myocardium Damage by Regulating AKT-Mediated Oxidative Stress, Cardiac Remodeling, and Inflammation

**DOI:** 10.3390/ijms231710146

**Published:** 2022-09-04

**Authors:** Pei-Ling Hsieh, Pei-Ming Chu, Hui-Ching Cheng, Yu-Ting Huang, Wan-Ching Chou, Kun-Ling Tsai, Shih-Hung Chan

**Affiliations:** 1 Department of Anatomy, School of Medicine, China Medical University, Taichung 404, Taiwan; 2 Department of Anatomy, School of Medicine, Chung Shan Medical University, Taichung 402, Taiwan; 3 Department of Physical Therapy, College of Medicine, National Cheng Kung University, Tainan 701, Taiwan; 4 Institute of Allied Health Sciences, College of Medicine, National Cheng Kung University, Tainan 701, Taiwan; 5 Department of Internal Medicine, College of Medicine, National Cheng Kung University Hospital, National Cheng Kung University, Tainan 704, Taiwan

**Keywords:** doxorubicin, dapagliflozin, cardiotoxicity, Akt

## Abstract

Doxorubicin (Dox) is a commonly used anthracycline chemotherapy with a side effect of cardiotoxicity, which may increase the risk of heart failure for cancer patients. Although various studies have demonstrated the cardioprotective property of dapagliflozin (DAPA), a sodium-glucose cotransporter 2 inhibitor, the detailed mechanism underlying its effect on Dox-induced cardiomyopathy is still limited. In this study, we showed that DAPA induced the activation of AKT/PI3K signaling in cardiac myoblast H9c2 cells following Dox treatment, leading to the upregulation of antioxidant HO-1, NQO1, and SOD, as well as an improved mitochondrial dysfunction via Nrf2. In addition, the reduced oxidative stress resulted in the downregulation of hypertrophy (ANP and BNP) and fibrosis (phospho-Smad3, collagen I, fibronectin, and α-SMA) markers. Furthermore, the inflammatory IL-8 concentration was inhibited after DAPA, possibly through PI3K/AKT/Nrf2/p38/NF-κB signaling. Moreover, our results were validated in vivo, and echocardiography results suggested an improved cardiac function in DAPA-receiving rats. In summary, we demonstrated that the administration of DAPA could mitigate the Dox-elicited cardiotoxicity by reducing oxidative stress, mitochondrial dysfunction, fibrosis, hypertrophy, and inflammation via PI3K/AKT/Nrf2 signaling.

## 1. Introduction

Doxorubicin (Dox) is a commonly used anthracycline chemotherapeutic regimen. Although it is efficacious in tumor regression, the cardiotoxic side effects limit patients’ treatment and overall survival. It has been reported that the incidence of anthracycline-induced cardiotoxicity was around 9% of patients, and 98% of them developed cardiotoxicity within the first year after the completion of therapy [1]. The presence of inflammation, interstitial fibrosis, left ventricular ejection fraction (LVEF) reduction, or even heart failure was detected following the treatment of anthracycline-based chemotherapy [1,2]. As such, it is imperative to develop proper management strategies for Dox-receiving patients and decrease the occurrence of impaired cardiac function and adverse outcomes. 

Dapagliflozin (DAPA), an inhibitor of sodium–glucose cotransporter 2 (SGLT2), has been shown to mitigate the risk of heart failure or cardiovascular death in patients with a reduced LVEF [3] or type II diabetes [4]. Multiple contributors have been considered to implicate the Dox-induced cardiomyopathy, such as the formation of reactive oxygen species (ROS), induction of apoptosis, or aberrant signaling pathways [5]. It has been shown that DAPA treatment markedly suppressed the Dox-induced apoptosis and ROS accumulation in cardiomyocytes and improved cardiac fibrosis and cardiac function in normal and diabetic rats through the downregulation of ER stress or restoration of STAT3 [6,7]. However, the detailed molecular mechanisms underlying the protective effects of DAPA on Dox-elicited cardiomyopathy remain largely unknown. 

AKT/PKB is a serine/threonine protein kinase that serves as a critical regulator of cell survival. Numerous studies have suggested that AKT is involved in Dox-triggered oxidative stress and cardiotoxicity [8,9]. In addition, DAPA has been shown to exert its cardioprotective property via the AKT pathway [10]. Accordingly, we aimed to examine whether the treatment of DAPA ameliorated the Dox-induced cardiomyopathy through the regulation of AKT and associated signaling pathways. To this end, we utilized cardiac myoblast H9c2 cells and assessed the AKT phosphorylation in response to Dox stimulation. Subsequently, we dissected how DAPA inhibited ROS production, mitochondrial dysfunction, fibrosis, and inflammation in vitro and in vivo through AKT signaling.

## 2. Results

### 2.1. DAPA Reverses the Dox-Induced Repression of HO-1 and NQO1 via PI3K/AKT/Nrf2 Axis in H9c2 Cells

**Figure 1:**
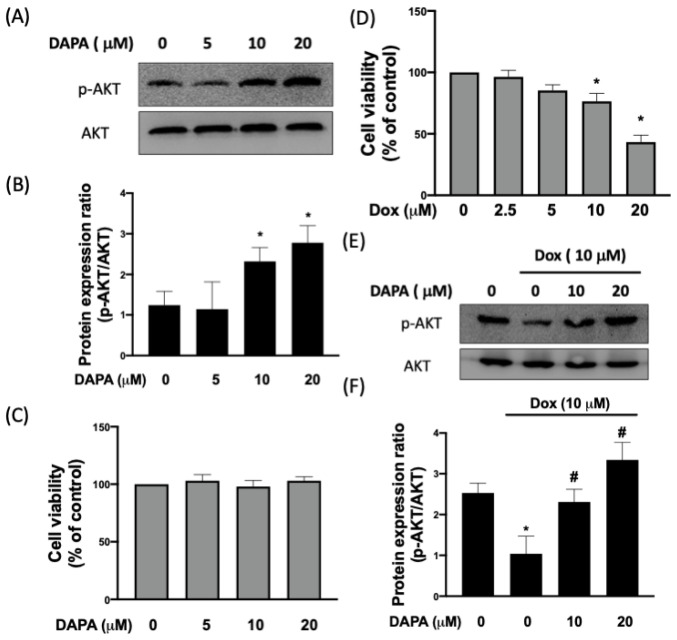
Treatment of DAPA restores the Dox-inhibited phosphorylation of AKT. Representative Western blot images (**A**) and densitometric analysis (**B**) of phosphorylated and total AKT levels in H9c2 cells treated with various concentrations of DAPA. Cell viability of H9c2 cells after DAPA treatment (**C**). The dosage-dependent manner of Dox exposure (**D**). Representative Western blot images (**E**) and densitometric analysis (**F**) of phosphorylated and total AKT levels in Dox-stimulated H9c2 cells following DAPA co-administration were shown. Results are shown as means ± SD of three independent experiments. Statistical analysis was performed using one-way ANOVA with a Bonferroni post hoc test. [* *p* < 0.05 compared to the control group; # *p* < 0.05 compared to the Dox-only group].

As shown in Figure 1A,B, the expression of phosphorylated AKT was increased dose-dependently in cardiac myoblast H9c2 cells following various concentrations of DAPA treatment (0–20 μM). In addition, we confirmed that DAPA treatment has no effects on cell viability or proliferation (Figure 1C). Dox exposure reduced cell viability in a dosage-dependent manner. Stimulation of 10μM Dox for 24 h decreased cell viability by more than 50% in H9c2 cells. Thus, this dosage was used for further investigations (Figure 1D). In addition, we observed that the phosphorylation of AKT was downregulated in response to Dox, whereas the administration of DAPA resulted in a dose-dependent reversal of AKT phosphorylation (Figure 1E,F). 

**Figure 2:**
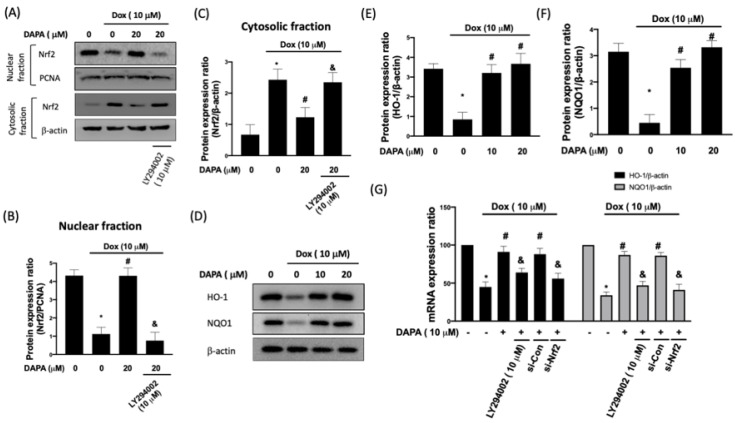
**DAPA administration prevents the Dox-inhibited Nrf2 nuclear translocation and expression of HO-1 and NQO1**. Western blot images (**A**), densitometric analysis of nuclear fraction (**B**), and cytosolic fraction (**C**) of Nrf2 in Dox-treated H9c2 cells were shown. Representative Western blot images (**D**), densitometric analysis of HO-1 (**E**) and NQO1 (**F**) in Dox-stimulated cells with or without DAPA treatment were revealed. Expression levels of HO-1 and NQO1 gene in Dox-treated cells with or without DAPA treatment were checked (**G**). LY294002 (a PI3K inhibitor) and si-Nrf2 were used in some experiments. Results are shown as means ± SD of three independent experiments. Statistical analysis was performed using one-way ANOVA with a Bonferroni post hoc test. [* *p* < 0.05 compared to the control group; # *p* < 0.05 compared to the Dox-only group; & *p* < 0.05 compared to Dox + DAPA group].

Nuclear factor erythroid-2 related factor 2 (Nrf2) has been shown to act as an endogenous suppressor of Dox-induced cardiotoxicity [11], and the phosphatidylinositol 3 kinase (PI3K)/AKT pathway is essential for Nrf2 activation [12]. With the treatment of Dox, Nrf2 was sequestered in the cytoplasm, while nuclear accumulation of Nrf2 was observed again when DAPA was co-treated (Figure 2A–C). Nevertheless, this effect was abrogated following the inhibition of PI3 kinase-dependent AKT phosphorylation using LY294002 (Figure 2A–C), suggesting that PI3K/AKT signaling contributed to Dox-triggered Nrf2 nuclear translocation. 

In line with the results of Nrf2, the expression levels of heme oxygenase-1 (HO-1) and NAD(P)H: quinone oxidoreductase (NQO1), two downstream antioxidant genes of Nrf2 [13], were downregulated in Dox-treated H9c2 cells (Figure 2D–F). However, DAPA administration reversed the expression of HO-1 and NQO1 (Figure 2D–F). In order to validate that the alteration of HO-1 and NQO1 was due to the PI3K/AKT/Nrf2 axis, gene expression was assessed in cells treated with LY294002 or transfected with si-Nrf2. As expected, DAPA was able to reverse the Dox-inhibited expression of HO-1 and NQO1, while the inhibition of PI3K/AKT or Nrf2 blocked its effect (Figure 2G). These results suggested that the reduced expression of HO-1 and NQO1 following Dox treatment could be reversed by DAPA through PI3K/AKT/Nrf2 axis.

### 2.2. DAPA Restores the DOX-Induced Downregulation of Antioxidant Capacity and Mitochondrial Dysfunction in H9c2 Cells

**Figure 3:**
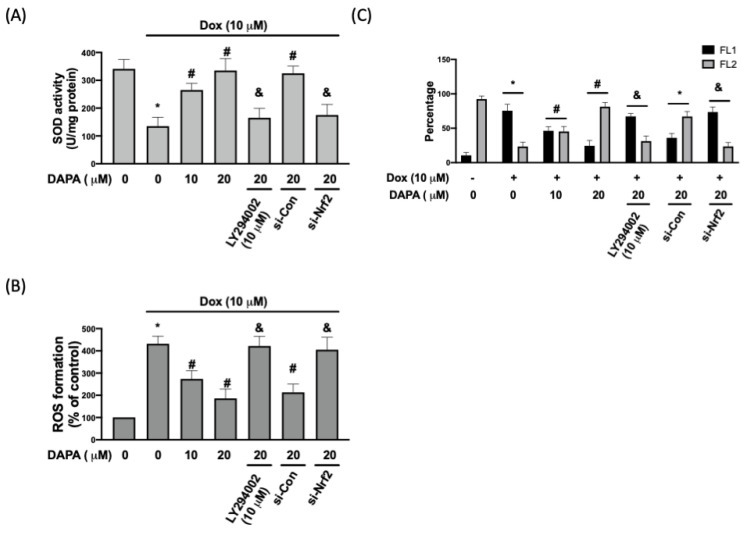
**DAPA diminishes Dox-induced oxidative stress and mitochondrial damage.** (**A**) SOD activity, (**B**) ROS formation, and (**C**) percentage of cells expressing JC-1 monomers (green fluorescence; FL1) and JC-1 aggregates (red fluorescence; FL2) in Dox-treated cells with or without DAPA treatment were assessed. Results are shown as means ± SD of three independent experiments. Statistical analysis was performed using one-way ANOVA with a Bonferroni post hoc test. [* *p* < 0.05 compared to the control group; # *p* < 0.05 compared to the Dox-only group; & *p* < 0.05 compared to Dox + DAPA group].

Aside from HO-1 and NQO1, superoxide dismutase (SOD) is another antioxidative enzyme that mediates cardioprotection under the regulation of Nrf2 [14]. We showed that the Dox-inhibited SOD activity was rescued by the treatment of DAPA, and this effect was mitigated when PI3K/AKT signaling or Nrf2 was suppressed (Figure 3A). ROS production was altered in accordance with the changes in SOD activity. The Dox-induced ROS formation was suppressed by DAPA administration, whereas the blockage of the PI3K/AKT/Nrf2 axis prevented this inhibitory activity (Figure 3B). It is known that mitochondrial dysfunction is involved in various cardiovascular-related diseases and that ROS accumulation can lead to mitochondrial dysfunction [15]. One of the methods used to monitor mitochondrial membrane potential is to use the membrane-permeant JC-1 dye, which aggregates in the mitochondria and forms red fluorescent agglomerates in healthy cells. In Figure 3C, we observed a reduced percentage of cells expressing JC-1 red fluorescence (FL2) after Dox treatment. With the increased DAPA, the percentage of JC-1 red fluorescence (FL2) was gradually elevated. Nevertheless, this protective effect was not observed in cells treated with LY294002 or transfected with si-Nrf2 as there were more cells expressing green fluorescence (FL1) in these groups (Figure 3C). Collectively, these findings indicated that DAPA may diminish the generation of oxidative stress and correct mitochondrial dysfunction in Dox-treated cells.

### 2.3. DAPA Suppresses the DOX-Elicited Cardiac Remodeling

**Figure 4:**
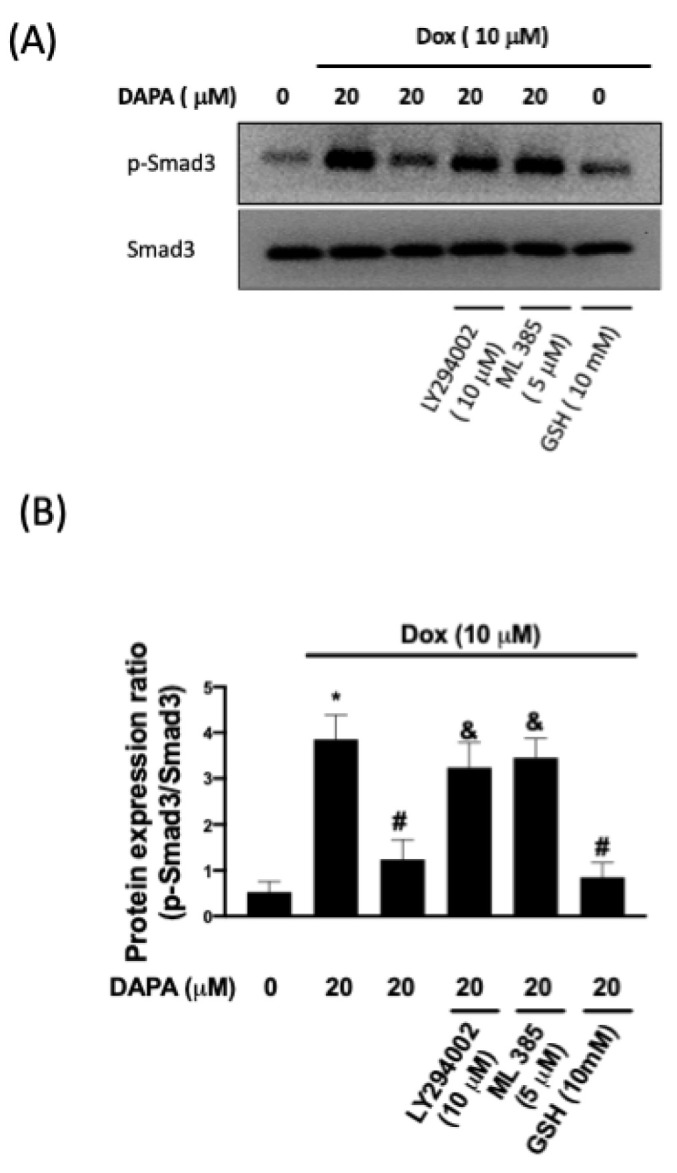
**Phosphorylation of Smad3 in Dox-treated cells.** Representative Western blot images (**A**) and densitometric analysis (**B**) of phospho-Smad3 and total Smad3 in H9c2 cells exposed to Dox stimulation with or without DAPA treatment were shown. LY294002 (a PI3K inhibitor), ML385 (a Nrf2 inhibitor), and GSH (an antioxidant) were used in some experiments. Results are shown as means ± SD of three independent experiments. Statistical analysis was performed using one-way ANOVA with a Bonferroni post hoc test. [* *p* < 0.05 compared to the control group; # *p* < 0.05 compared to the Dox-only group; & *p* < 0.05 compared to Dox + DAPA group].

Oxidative stress and mitochondrial dysfunction have been known to be associated with various cardiac pathologies, such as hypertrophy and fibrosis [16]. Western blot results showed that Dox increased the phosphorylation of Smad3 in H9c2 cells, which was inhibited by DAPA treatment (Figure 4A,B). Apart from LY294002, we showed that the application of the Nrf2 inhibitor (ML385) abolished the inhibitory effect of DAPA on the phosphorylation of Smad3 (Figure 4A,B). Moreover, our data demonstrated that the expression of phosphorylated Smad3 was downregulated in cells co-treated with Dox and antioxidant glutathione (GSH).

**Figure 5:**
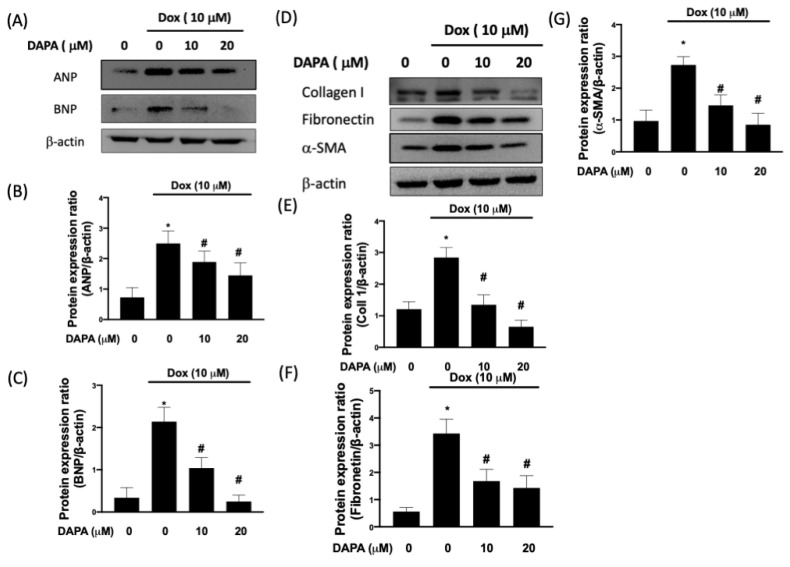
DAPA mitigates the expression of cardiac fibrosis markers in Dox-stimulated cells. Representative Western blot images (**A**) and densitometric analysis of ANP (**B**) and BNP (**C**) in Dox-treated H9c2 cells with various concentrations of DAPA treatment were shown. Representative Western blot images (**D**), densitometric analysis collagen I (**E**), fibronectin (**F**), and α-SMA (**G**) in Dox-treated H9c2 cells with various concentrations of DAPA treatment were disclosed. Results are shown as means ± SD of three independent experiments. Statistical analysis was performed using one-way ANOVA with a Bonferroni post hoc test. [* *p* < 0.05 compared to the control group; # *p* < 0.05 compared to the Dox-only group].

Atrial natriuretic peptide (ANP) and brain (B-type) natriuretic peptide (BNP) are two crucial cardioprotective hormones and are found to be elevated during cardiac remodeling, which is characterized by left ventricular hypertrophy and cardiac fibrosis [17]. In Figure 5A–C, the Dox-induced upregulation of ANP and BNP was dose-dependently suppressed by DAPA treatment. In accordance with the abovementioned results, we showed that various fibrosis indicators, including type I collagen, fibronectin, and α-smooth muscle actin (α-SMA), were increased in Dox-treated cells but not in cells receiving both Dox and DAPA (Figure 5D–G). Taken together, our results demonstrated that the administration of DAPA enables cells to resist adverse cardiac remodeling.

### 2.4. DAPA Ameliorates the DOX-Stimulated Cardiac Inflammation in H9c2 Cells

**Figure 6:**
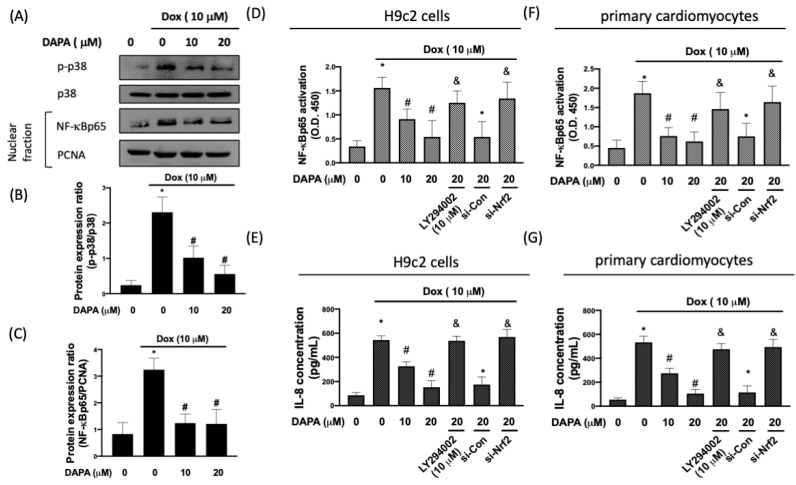
**DAPA treatment suppresses the production of Dox-induced inflammatory mediators.** Representative Western blot images (**A**) and densitometric analysis of phospho-p38 (**B**) and NF-κB p65 (**C**) in Dox-stimulated H9c2 cells with various concentrations of DAPA treatment were shown. NF-κB p65 activation (**D**) and IL-8 production (**E**) in Dox-treated H9c2 cells with various concentrations of DAPA treatment were examined by ELISA assay. LY294002 (a PI3K inhibitor) and si-Nrf2 were used in some experiments. The finding of NF-κB p65 activation (**F**) and IL-8 production (**G**) were conducted in primary cardiomyocytes. Results are shown as means ± SD of three independent experiments. Statistical analysis was performed using one-way ANOVA with a Bonferroni post hoc test. [* *p* < 0.05 compared to the control group; # *p* < 0.05 compared to the Dox-only group; & *p* < 0.05 compared to Dox + DAPA group].

It is well known that inflammation occurs as a consequence of oxidative stress, and the activation of the p38 mitogen-activated protein kinase (MAPK)/nuclear factor-κB (NF-κB) pathway has been proven to contribute to Dox-induced inflammation in H9c2 cells [18]. Here, we showed that the administration of DAPA mitigated the Dox-elicited upregulation of phosphorylated p38 and nuclear NF-κB p65 (Figure 6A–C). Likewise, the activation of NF-κB p65 was decreased in Dox-treated cells when DAPA was co-treated (Figure 6D). In addition, we showed that the repression of PI3K/AKT signaling or Nrf2 obstructed this suppressive effect on NF-κB p65 activation by DAPA (Figure 6D).

A higher level of interleukin (IL)-8 has been shown to be implicated in the development of heart failure [19], and our previous work has demonstrated that Dox-induced an increase in the IL-8 of cardiac cells [20]. We showed that the downregulation of the PI3K/AKT/Nrf2 axis blocked the inhibitory property of DAPA on IL-8 production in Dox-stimulated cells (Figure 6E). The findings of NF-κB p65 activation (Figure 6F) and IL-8 production (Figure 6G) were further confirmed in primary cardiomyocytes. Given that NF-κB p65 activation is associated with the transcriptional activation of IL-8 [21], our data demonstrated that DAPA was able to downregulate the secretion of IL-8 in cardiac cells in response to Dox through PI3K/AKT/Nrf2/p38/NF-κB signaling.

### 2.5. Cardioprotective Effect of DAPA in Dox-Injected Rats

**Figure 7:**
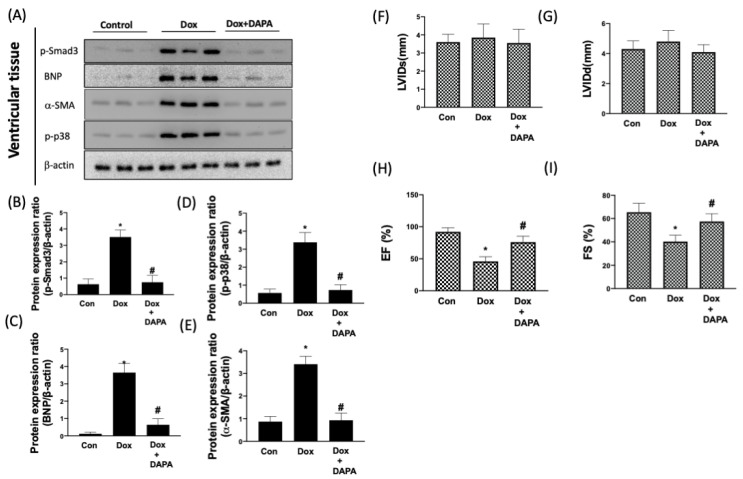
**DAPA protects heart function in response to Dox treatment.** Representative Western blot images (**A**) and densitometric analysis of phospho-Smad3 (**B**), BNP (**C**), α-SMA (**D**), and phospho-p38 (**E**) in control (CON) group, Dox only group, and combination treatment of Dox and DAPA (Dox + DAPA) group were shown. (**F**) Left ventricular internal dimension at end-diastole (LVIDd), (**G**) left ventricular internal dimension at end-systole (LVIDs), (**H**) ejection fraction (EF), and (**I**) fractional shortening (FS) of the heart were measured using echocardiography. Results are shown as means ± SD of three independent experiments. Statistical analysis was performed using one-way ANOVA with a Bonferroni post hoc test. [* *p* < 0.05 compared to the control group; # *p* < 0.05 compared to the Dox-only group].

In order to validate the results in vivo, the aforementioned parameters and Dox-caused cardiac dysfunction were examined after DAPA administration. We showed that the expression levels of phosphorylated Smad3, BNP, α-SMA, and phosphorylated p38 were all increased in Dox-treated cells, whereas the administration of DAPA prevented the elevation of these factors (Figure 7A–E). For the cardiac function test, data from echocardiography showed no significant differences in left ventricular internal dimension at end-diastole (LVIDd) and left ventricular internal dimension at end-systole (LVIDs) in each group (Figure 7F,G). However, the left ventricular ejection fraction (LVEF) and left ventricular fractional shortening (LVFS) were markedly decreased in the Dox-receiving rats. Nonetheless, the cardiotoxicity of Dox appeared to be attenuated following co-treatment with DAPA, as it prevented these two values from dropping (Figure 7H,I). These results confirmed that the administration of DAPA ameliorated the myocardial dysfunction and unfavorable molecular changes that may lead to cardiac impairment.

## 3. Discussion

**Figure 8:**
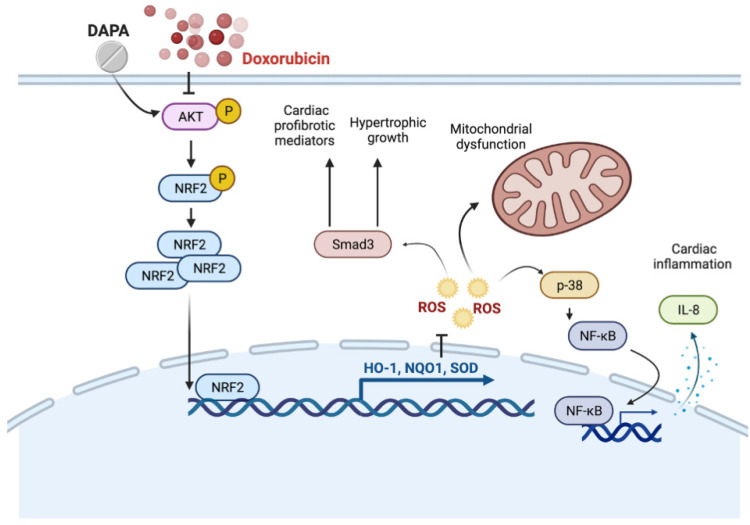
**Schematic diagram of the major study findings.** This study presents that DAPA reduces Dox-caused myocardial damage by regulating AKT-mediated oxidative stress, cardiac remodeling, and inflammation.

Despite Dox being a promising and frequently used chemotherapeutic drug, its fatal cardiotoxic effects have still prevailed in the fight against cancer since the late 1960s. The intense interest in deciphering the pathophysiology of Dox-induced cardiomyopathy has been aroused, and multiple studies have shown the potential mechanisms, such as increased death receptors-mediated apoptosis in cardiomyocytes [22], the collagen production of cardiac fibroblasts [23], or the alteration of mitochondrial dynamics [24] in response to Dox treatment. While a large body of knowledge is available on the cardioprotective effects of DAPA, the precise molecular mechanisms through which DAPA eventually rescued the Dox-induced cardiac damage are much less clear. In the present study, we demonstrated that the administration of DAPA downregulated oxidative stress accumulation and mitochondrial dysfunction through the restoration of antioxidant capacity (e.g. HO-1, NQO1, and SOD) via the PI3K/AKT/Nrf2 axis (Figure 8).

Multiple studies have demonstrated the antioxidant capacity of DAPA in various types of tissues, such as kidney [25], brain [26], liver [27,28], and lung [29]. In agreement with our findings, it has been shown that DAPA increased the level of antioxidant GSH in the liver of diabetic rats [28] and activated the Nrf2/HO-1 pathway in cholesterol-treated mice hepatocytes [27], as well as in the kidney of diabetic mice [25]. Moreover, it has been demonstrated that the administration of DAPA downregulated the production of lipid peroxides and counteracted the changes in the DJ-1 (Parkinson protein 7; PARK7)/Nrf2 pathway in rotenone-elicited neuronal injury [26]. Likewise, Arab et al. showed that DAPA activated the PI3K/AKT/GSK-3β signaling, which reduced the ROS-dependent neuronal apoptosis in the rotenone-induced Parkinson’s disease rat model [26]. On the other hand, it is well-known that defective mitochondria increased the production of excessive ROS. In H9c2 cells, it has been shown that Dox induced morphological alterations in mitochondrial protein structures [30] and mitochondrial depolarization following nuclear p53 activation [31]. In addition, GTPases that are implicated in the balance between the fission and fusion of mitochondria were affected by Dox treatment as well [24], and the administration of DAPA has been shown to normalize the expression of these factors, the mitochondrial respiratory ratio, and the mitochondrial DNA copy number [32]. Our previous work also demonstrated that DAPA prevented the hypoxia/reoxygenation-induced abnormality of the mitochondrial membrane potential and mitochondrial DNA copy number through AMPK/PKC/NADPH oxidase signaling [33]. In the present study, we showed that the impaired mitochondrial membrane potential was restored through Nrf2 activation. Altogether, it is possible that DAPA ameliorated Dox-induced mitochondrial dysfunction through the normalization of mitochondrial fission/fusion enzymes and downregulation of excessive oxidative stress.

Another benefit of DAPA to diminish the Dox-induced cardiotoxicity is its anti-fibrotic property. The altered cardiac mitochondrial bioenergetics have been shown to participate in the accumulation of oxidative stress, which leads to cardiac apoptosis [34]. In the heart of Dox-injected mice, an increased apoptosis and left ventricular collagen deposition were observed [35]. Coincidental with the improvement of mitochondrial dysfunction after DAPA treatment, the reduced expression of fibrosis markers was shown in the current study. Given that cardiac fibrosis is often a consequence of apoptosis, we postulated that the normalization of mitochondrial dynamics by DAPA downregulated cardiac apoptosis and the subsequent fibrosis. In addition, we showed that the phosphorylation of Smad3 was inhibited in cells co-treated with Dox and antioxidant GSH, suggesting that the antioxidant activity of DAPA suppressed the Dox-induced fibrotic response. Apart from cardiomyocyte apoptosis, Dox has been found to increase the viability of cardiac fibroblasts with increased collagen production [23]. It has been demonstrated that DAPA treatment markedly repressed the expression of activated fibroblast marker (α-SMA) and fibrosis marker (e.g. collagen, vimentin, and TGF-β/Smad signaling) [36]. Hence, it is plausible that the administration of DAPA prevented the Dox-elicited activation of cardiac fibroblasts.

By using low-dose Dox to provoke perivascular fibrosis without causing apoptosis, Tanaka et al. showed that damaged mitochondria served as damage-associated molecular patterns (DAMPs) to initiate an inflammatory response, which promoted cardiac fibrosis and adverse cardiac remodeling [37]. Our data showed that DAPA administration downregulated the inflammatory response through PI3K/AKT/Nrf2/p38/NF-κB signaling, indicating that the anti-inflammatory effect may be another rationale for the anti-fibrosis property of DAPA. In addition, we showed that both ANP and BNP were upregulated in H9c2 cells incubated with Dox, and LVEF and LVFS were markedly reduced in the Dox-receiving rats. This finding was consistent with a previous study of cancer patients showing that plasma levels of ANP, NT-pro-ANP (the active form of ANP), and BNP were all increased after Dox treatment [38]. Moreover, there was a negative correlation between the decrease in FS and the increase in plasma NT-pro-ANP and plasma BNP [38]. In the present study, we showed that DAPA was able to inhibit the Dox-induced upregulation of these natriuretic peptides, particularly BNP. In line with these results, DAPA has been proven to significantly diminish NT-pro-BNP, the risk of worsening heart failure, and death in subjects with heart failure with reduced LVEF [39]. Since various inflammatory mediators upregulated the expression of BNP that was secreted by cardiac fibroblasts [40], it is reasonable to speculate that the favorable effects of DAPA on the suppression of these natriuretic peptides may be due to its anti-inflammatory property and inhibitory effect on fibroblast activation. 

IL-8 has been shown to be a significant predictor of adverse outcomes in patients with chronic heart failure instead of other inflammatory cytokines [41]. NF-κB p65 activation is associated with an increase in IL-8 [21], and our results revealed that both Dox-stimulated NF-κB p65 activation and IL-8 production were inhibited by DAPA via the PI3K/AKT/Nrf2 cascade. This finding supports the antioxidant capacity of the DAPA diminished inflammatory response. It has been shown that phosphorylated AKT was markedly reduced in the heart of Dox-treated mice [35] and rats [20], and that the upregulation of cardiac PI3K/AKT signaling ameliorated the Dox-induced cardiomyopathy in vivo [8]. Various studies have demonstrated that the modulation of the PI3K/AKT axis by DAPA treatment improved cardiac function in murine models of myocardial infarction [42] and type I diabetes [10]. Consistent with these findings, we showed that the restoration of AKT following DAPA administration enhanced the parameters of cardiac function (EF and FS) in Dox-receiving rats. To substantiate the in vitro findings, we scrutinized the anti-fibrosis and anti-inflammatory effects of DAPA by measuring the expression of phospho-Smad3, BNP, α-SMA, and phosphorylated p38 in left ventricular tissues. The results of the protein expression of these markers in concert with the assessment of cardiac function suggested that DAPA lessens the Dox-induced cardiomyopathy through its anti-fibrosis and anti-inflammatory effects mediated by the PI3K/AKT/Nrf2 cascade.

This present study has several limitations. 1. Only male animals were used in this study. The female animals may be more sensitive to Dox and mimic clinical status. 2. We did not measure physiological parameters such as heart rate and blood pressure. These parameters are critical for clinical patients with cardiotoxicity. All of these issues will be our future direction of study. 

In conclusion, this study reports that DAPA treatment protects against Dox-caused cardiotoxicity and myocardium injury by modulating the PI3K/AKT/Nrf2 pathway, thereby enhancing antioxidant capacity and inhibiting mitochondrial dysfunction. The results from this study provide new information for clinical management in cancer patients. 

## 4. Materials and Methods

### 4.1. Cell Culture and In Vivo Model

The myoblasts cells, H9c2, were bought from the American Type Culture Collection (ATCC; Catalog number: CRL-1446). H9c2 cells were cultured with high-glucose Dulbecco’s modified Eagle’s medium (DMEM) combined with 10% FBS. The penicillin (50 IU/mL) and streptomycin (50 µg/mL) were added during cell culture. A 0.25% (*w*/*v*) Trypsin-EDTA was selected to passage cells. H9c2 cells were cultured in humidified air with 5% CO_2_ at 37 °C. The seeding density in a 10 cm dish was 1 × 10^6^ cells. After 24 h of cell seeding, further investigation was performed. The cells used for investigations in this study were less than 20 passages. For the animal study, the experimental procedures were approved by the Ethics Committee of Cheng Kung University, Tainan, Taiwan, and conducted in accordance with the ‘Guide for Care and Use of Laboratory Animals’. (Approval No. 109317). Three groups of Sprague Dawley (SD) rats underwent the following treatments: control (saline), Dox and Dox plus DAPA treatment, *n* = 18 (6 in each group). Dox was dissolved in saline. Dox was administered at 3 mg/kg body weight (BW) via four weekly intraperitoneal (i.p.) injections, giving a cumulative dose of 12 mg/kg BW. Control rats received three i.p. injections of 0.9% saline on alternative days. In DAPA treatment animals, the DAPA was administered by the oral route with the dosage 0.1 mg/kg per day. DAPA was dissolved in 60% propylene glycol and was given daily by gavage for five days for four weeks until sacrifice. Before sacrifice, echocardiography was performed to assess cardiac function. Anesthesia was induced by placing animals in a box with 5% isoflurane for 5 min and then maintained in 1.0–1.5% isoflurane through a facemask. Isoflurane-anesthetized animals were placed in a supine position. Echocardiographic data were collected by a Vevo 770 microimaging system with a 25 MHz probe (VisualSonics, Toronto, ON, Canada). Parameters were collected based on the M-mode and two-dimensional images obtained in the parasternal long and short axis views at the level of the papillary muscles.

### 4.2. Reagents 

The fetal bovine serum FBS and EDTA were obtained from Gibco (NY, USA). The 2′,7′-Dichlorofluorescein diacetate (DCFHDA), 5,58,6,68-tetraethylbenzimidazolcarbocyanine iodide (JC-1), doxorubicin (Dox), LY294002, ML 385, and glutathione (GSH) were bought from Sigma-Aldrich (St. Louis, MO, USA). The anti-AKT, anti-phospho-AKT, anti-β-actin, anti-Nrf2, anti-PCNA, anti-HO-1, anti-NQO1, and anti-NFkBp65 antibodies were obtained from Cell Signaling Technology (Danvers, MA, USA). The anti-BNP, anti-ANP, anti-Smad3, anti-phospho-Smad3, anti-p38, anti-phospho-p38, anti-α-SMA, anti-fibronectin, and anti-collagen 1 antibodies were obtained from Santa Cruz Biotechnology (Dallas, CA, USA). The secondary antibodies with HRP conjugated were bought from Sigma-Aldrich (St. Louis, MO, USA).

### 4.3. Cell Viability

The MTT assay was used to assess cell viability. Cells were incubated for 1 h in DMEM containing 0.5 mg/mL MTT. The incubation buffer was removed, and the blue MTT–formazan product was extracted with dimethyl sulfoxide (DMSO). The absorbance of the formazan solution was read spectrophotometrically at 570 nm.

### 4.4. Primary Culture of Cardiomyocytes

Newborn rats were sacrificed by cervical dislocation within 24 h. The rat ventricles were washed with iced PBS twice. The tissues were minced into small pieces and digested with 0.25% pancreatin and 0.1% collagenase in humidified air with 5% CO2 at 37 °C for 15 min. Lysates were obtained by centrifugation at 1300 rpm for 15 min. The pellet was resuspended in DMEM/F-12 with 10% FBS and penicillin.

### 4.5. Western Blotting Assay

Total protein was isolated from H9c2 cells or the left ventricle cardiac tissue. After sacrifice, the left ventricle tissues were washed three times with iced 1xPBS buffer, and then 100 mg of tissue was collected for homogenization. The homogenates were centrifuged at 12,500× *g* rpm for 20 min, and the supernatant was collected for Western blotting assay. The RIPA was used to isolate total protein from H9c2 cells. The proteins were transferred to a PVDF membrane after the proteins were separated by electrophoresis on sodium dodecyl sulfate polyacrylamide gels. Then, the membranes were blocked by buffer at room temperature for two hours. Then, the membranes were incubated with primary antibodies at 4 °C for twenty hours, followed by hybridization with HRP-conjugated secondary antibodies for one hour. The intensities were quantified by densitometric analysis using ImageJ.

### 4.6. RNA Isolation and Analysis

RNA was isolated using TRIzol reagent (Invitrogen, Carlsbad, CA, USA) according to the manufacturer’s instructions. Oligonucleotide specificity was determined by a homology search within the genome (BLAST, National Center for Biotechnology Information, Bethesda, MD, USA) and confirmed by dissociation curve analysis. The oligonucleotide sequences were: β-actin; F:5’-CCCTGGCTCCTAGCACCAT-3’; R:5’-GATAGAGCCACCAATCCAATCCACACA-3’. HO-1; F: 5’- CGTGCAGAGAATTCTGAGTTC-3’; R: 5’-AGACGCTTTACGTAGTGCTG-3’. NQO1; F: 5′-CTGCCATCATGCCTGACTAA-3′; R: 5’-TGCAGATGTACGGTGTGGAT-3’. The real-time PCR was conducted with SYBR Green in an ABI 7000 sequence detection system (Applied Biosystems) according to the manufacturer’s guidelines.

### 4.7. Investigation of Mitochondrial Membrane Potential

The mitochondrial membrane potential (ΔΨm) was investigated via the lipophilic cationic probe fluorochrome 5, 58, 6, 68 tetraethylbenzimidazolcarbocyanine iodide (JC-1). The JC-1 dye presents either as a green fluorescent (FL-1) monomer at depolarized membrane potentials or as a red fluorescent (FL-2) J-aggregate at hyperpolarized membrane potentials. After treating the cells with Dox with or without DAPA for twenty-four hours, H9c2 cells were exposed to 5 μM JC-1. H9c2 cells were studied by flow cytometry after a twenty-five-minute incubation at 37 °C.

### 4.8. Cytoplasmic and Nuclear Protein Extraction and NF-kB Activity

Nuclear and cytosolic extracts were isolated with a NE-PER™ Nuclear and Cytoplasmic Extraction kit (Catalog number: 78833, Thermo Fisher Scientific, Waltham, MA, USA). The nuclear extracts (supernatants) were stored at −80 °C until use. The NF-kB p65 Transcription Factor Assay Kit (Catalog number: ab133112, Abcam, Cambridge, UK) was used for testing the activity of NF-kB.

### 4.9. IL-8 Concentration and SOD Activity

IL-8 concentration and SOD activity were investigated by commercial kits according to the manufacturer’s instructions. In brief, the antibody in the coating buffer was added to individual wells and maintained for two hours at RT. Next, the coating solution was removed, and wells were washed with PBS-0.05% Tween-20 three times. The blocking buffer was loaded in each well for sixteen minutes at 37 °C. An aliquot of 50 µL of diluted antibody was added to each well for sixteen minutes. After that, an aliquot of 50 µL of conjugated secondary antibody was added to each well for sixteen minutes of incubation. The O.D. 450 nm was read. The IL-8 kit was bought from R&D (Minneapolis, MN, USA). The SOD activity was purchased from Biovision (San Francisco, CA, USA).

### 4.10. Investigation of ROS Production

The ROS generation in the H9c2 cells was determined using DCFHDA. H9c2 cells were pre-treated with or without DAPA for two hours. Next, cells were stimulated to Dox for twenty hours. After removing the medium from the wells, cells were washed by PBS twice and incubated with 10 µM DCFHDA for twenty-five minutes. The fluorescence intensity was investigated by flow cytometry.

### 4.11. Transfection with Small Interfering RNA

The Nrf2 siRNA and negative control siRNA were obtained from Dharmacon. Forty-eight hours after transfection, H9c2 cells were treated with a reagent as indicated for further experiments. The knockdown efficiency was confirmed by Western blotting assay. 

### 4.12. Statistical Analyses

Statistical analysis was performed with Statistical Product and Service Solutions (SPSS) software version 11.0 (SPSS, Inc., Chicago, IL, USA). Results are expressed as the means ± standard deviation (SD). The Bonferroni correction was applied to correct multiple testing. ANOVA of variance was used to compare data among groups in experiments including three or more groups. A *p*-value < 0.05 was considered statistically significant.

## Data Availability

Not applicable.
